# Supplementary material for: Modeling glioblastoma heterogeneity as a dynamic network of cell states
Source: Mol Syst Biol. 2021 Sep 16;17(9):e10105. doi: 10.15252/msb.202010105 (PMC8444284; doi:10.15252/msb.202010105)
Supplement: Supplementary file 5 — Source Data for Figure 3 [file MSB-17-e10105-s001.zip › Figure3A_sourcedata/GSEA_3065/nosigdb_state3.GseaPreranked.1622718426028/TIISIG_PMID_32649888.html]

Details for gene set TIISIG\_PMID\_32649888[GSEA]

|  || Dataset | state3 |
| Phenotype | NoPhenotypeAvailable |
| Upregulated in class | na\_pos |
| GeneSet | TIISIG\_PMID\_32649888 |
| Enrichment Score (ES) | 0.6731024 |
| Normalized Enrichment Score (NES) | 1.9499525 |
| Nominal p-value | 0.0 |
| FDR q-value | 8.884502E-4 |
| FWER p-Value | 0.007 |
Table: GSEA Results Summary

  

Fig 1: Enrichment plot: TIISIG\_PMID\_32649888      
 Profile of the Running ES Score & Positions of GeneSet Members on the Rank Ordered List

  

| PROBE | GENE SYMBOL | GENE\_TITLE | RANK IN GENE LIST | RANK METRIC SCORE | RUNNING ES | CORE ENRICHMENT || 1 | GRIA2 |  |  | 38 | 0.460 | 0.1576 | Yes |
| 2 | PDGFRA |  |  | 115 | 0.326 | 0.2638 | Yes |
| 3 | SOX11 |  |  | 117 | 0.325 | 0.3782 | Yes |
| 4 | LSAMP |  |  | 307 | 0.241 | 0.4414 | Yes |
| 5 | COL11A1 |  |  | 684 | 0.173 | 0.4594 | Yes |
| 6 | BAMBI |  |  | 701 | 0.170 | 0.5173 | Yes |
| 7 | DNM3 |  |  | 783 | 0.159 | 0.5641 | Yes |
| 8 | ERBB3 |  |  | 899 | 0.149 | 0.6033 | Yes |
| 9 | DLL3 |  |  | 999 | 0.138 | 0.6407 | Yes |
| 10 | APOD |  |  | 1113 | 0.129 | 0.6731 | Yes |
| 11 | NFASC |  |  | 1688 | 0.093 | 0.6399 | No |
| 12 | PLP1 |  |  | 2047 | 0.074 | 0.6250 | No |
| 13 | RNF144A |  |  | 3368 | 0.031 | 0.4849 | No |
| 14 | TNS3 |  |  | 3372 | 0.031 | 0.4956 | No |
| 15 | PKP4 |  |  | 4677 | -0.003 | 0.3473 | No |
| 16 | SIRT2 |  |  | 4877 | -0.008 | 0.3272 | No |
| 17 | AMOTL2 |  |  | 5031 | -0.012 | 0.3139 | No |
| 18 | GNAI1 |  |  | 5194 | -0.016 | 0.3008 | No |
| 19 | FRZB |  |  | 5464 | -0.023 | 0.2781 | No |
| 20 | FGF12 |  |  | 5669 | -0.029 | 0.2650 | No |
| 21 | WASF1 |  |  | 6025 | -0.040 | 0.2385 | No |
| 22 | MYLK |  |  | 6330 | -0.050 | 0.2215 | No |
| 23 | CNP |  |  | 7852 | -0.158 | 0.1030 | No |
Table: GSEA details [plain text format]

  

Fig 2: TIISIG\_PMID\_32649888: Random ES distribution      
 Gene set null distribution of ES for **TIISIG\_PMID\_32649888**
